# Occupational Stress and Quality of Life among Health Professionals During the COVID-19 Pandemic

**DOI:** 10.2478/jccm-2022-0012

**Published:** 2022-08-12

**Authors:** Efstratios Vamvakas, Ioanna Kontogeorgou, Aggeliki Ntaountaki, Georgia Karkouli, Eleni Pisimisi, Eirini Karampekiou, Efstathios Politis, Iordana Moskofi, Dimitrios Konitopoulos, Eleni Dokoutsidou, Maria Grigoropoulou, Maria Theodorakopoulou, Apostolos Armaganidis

**Affiliations:** 1 Attikon General University Hospital, Athens, Greece; 2 University of West Attika, Athens, Greece; 3 Second Health Centre, Peristeri, Greece; 4 Department of Medicine, National and Kapodistrian University of Athens, Athens Greece

**Keywords:** occupational stress, depression, quality of life, healthcare professionals, COVID-19

## Abstract

**Introduction:**

Healthcare professionals, due to the nature of their work, have always experienced occupational stress, depression and low quality of life, which have been aggravated during the COVID-19 pandemic.

**Aim:**

A large-scale cross-sectional descriptive correlational study aimed to investigate the impact of the COVID-19 pandemic on Greek healthcare professionals’ psychological status and quality of life.

**Material and Methods:**

The study was conducted at “Attikon” General University Hospital and the 2nd Health Region in Athens, Greece. An assessment of anxiety and depression was carried out using the Zung’s Self-Rating Anxiety and Depression Scale (SAS/SDS). To assess the participants’ Quality of Life (QoL) the Short Form Survey-36 (SF-36) was used.

**Results:**

147 healthcare professionals were enrolled in the study. 70.7% experienced normal stress levels, 23.8% mild, 4.8% moderate and 0.7% severe. Mild depression was experienced by 34.7%, moderate by 10.2% and severe by 1.4%, with a 53.7% showing no depressive symptoms. Women experienced higher levels of anxiety and depression (p=0.001 & 0.001 respectively), and were 5.4 times more at risk to develop anxiety [Odds Ratio (OR) 5.357, 95% Confidence Interval (CI), 1.95-14.72: p=0.001] and 3.4 depression (OR, 3.365, 95% CI, 1.59- 7.12: p=0.002). Nurses and other professionals experienced higher stress and depression levels (p=0.004 & 0.040 respectively) than doctors. Participants reporting more exhaustion exhibited higher anxiety and depression levels (p=0.001). Compared to the pre-COVID-19 era, women (p=0.001), other health professionals (p=0.001) and those experiencing more physical burnout during COVID-19 (p=0.005) reported worse physical health. Anxiety and depression were negatively correlated with most sub scales of SF-36 except social functioning and bodily pain (p=0.001).

**Conclusions:**

Healthcare professionals’ QoL has been affected by the COVID-19 pandemic and they experience higher levels of anxiety and depression. There is a need to develop strategies to address the negative psychological impact of this pandemic on healthcare professionals.

## Introduction

Since December 2019, the global community has been facing a new infectious disease, COVID-19 [1]. In Greece, the first case was reported on 26^th^ February, 2020. On April 4^th^ of the same year, the “restructuring” of the health care services so as to manage the pandemic was announced [2]. However, healthcare professionals provided care to patients at increased risk of contracting the disease [1] using inadequate protocols and sometimes inappropriate personal protective equipment [3].

Such working conditions may adversely affect healthcare workers, particularly their mental health [4,5], due to the work overload, constant exposure to COVID-19 patients and the uncharted waters of this new situation [5,6]. Although the resulting psychological effects may subside within a few weeks, they can be of great significance since they involve a combination of emotional, cognitive, physical and social reactions [7]. The resulting stress, as a reaction to pressure, can in turn lead to mental disorders, such as anxiety and depression [8].

The factors which contribute to the development of physical and psychological fatigue during this pandemic include the few protective measures, occupational hazards and work-life balance. Moreover, healthcare professionals are often reluctant to return home due to fear of exposing their family members to the virus [9] and the feeling of being stigmatized and rejected, which results in emotional and physical exhaustion [1]. This burnout syndrome, by definition, refers to experiencing fatigue over extended time periods along with reduced motivation and interest in work, leading to a reduction in productivity. It derives from excessive effort in the workplace with limited opportunities for recovery. Intensive patient care, high mortality and inappropriate working conditions combined with a lack of time to adequately address patients’ needs are among the factors which contribute to high risks of exhaustion [10]. However, amidst this sudden global crisis, it is important for healthcare professionals to maintain their physical and psychological health [11].

The present large-scale cross-sectional descriptive correlational study aimed to assess healthcare workers’ stress and depression levels as well as their quality of life (QoL) and compare them to those before this crisis.

## Materials and methods

The present study was carried out in the Intensive Care Unit (ICU) of a COVID-19 clinic, the clinic itself and an Emergency Department (ER) of “Attikon” General University Hospital and the 2^nd^ Health Centre of Peristeri (HC) of the 2^nd^ Health Region in Greece (HRG), where COVID-19 cases were received. The study protocol was approved by the ethics committees of both bodies (no. 206/27-4-2020 & no.24607/28.04.2020 respectively). The nursing directors and supervisors were then informed of the purposes of the study.

To determine the adequate number of participants for this study the G* Power Version 3.1.9.6 was used the results of which showed that 84 participants is considered sufficient based on the following:

Test family= t-test, statistical test = means: Difference between two independent means (two groups), type of power analysis= A priori: Compute required sample size-given α, power, and effect size. Input parameters: tails (two), effect size d (0.8), α err prob(0.05), power 1-β err prob (0.95), allocation ratio N2/N1(1) = total sample size 84. The participants in the study were 147 healthcare professional, exceeding the required number of 84.

### Sample characteristics

All participants (n=147) were healthcare professionals (doctors, nurses, and auxiliary staff) working in the frontline wards of COVID-19. They were contacted in their workplace by the authors and after ensuring the confidentiality of their data and their anonymity, they were explained the purposes of the study and they provided their written informed consent to participate in the study.

### Questionnaires

Self-rating questionnaires were used to collect the socio-demographic, professional data and COVID-19 relevant data, which included gender, age, marital status, number of children, educational level, clinical experience, length of service a first-line health professional, average work hours per shift and satisfaction with personal protective equipment.

To assess stress, the Greek version of the Zung’s Self-Rating Anxiety Scale (SAS) was used. It was translated and standardized for its use in the Greek population by Samakouri et al in 2012 [12] with Cronbach alpha 0.897 and Intraclass Correlation Coefficient (ICC) regarding testing/retesting 0.913. Spearman’s rho, regarding validity, of SAS with Spielberger Greek Stress Scale STAI-Gr.-X -state was 0.767, STAI-GR.-X-trait 0.801 and with ZDRS 0.8.5. The authors stated that the Greek version of SAS has very satisfactory psychometric properties regarding its reliability and validity.

Depression was assessed using the Greek version of the Zung’s Self-Rating Depression Scale (SDS), which was translated and standardized by Fountoulakis et al in 2001 [13] who found its Sensitivity and Specificity exceeding 90.00 at 44/45 with Cronbach alpha equal to 0.09 and test-retest reliability Pearson’s r at 0.92, suggesting that the Greek version of SDS is suitable for clinical and research use in the Greek population. Both scales are rated by their raw score or by their index, which is obtained by dividing the raw score with the maximum score which is 80. The minimum score is 20, and a score below 50 (index 0.62) indicates absence of depression, 50-59 (index 0.62-0.74) mild depression, while a score 60-69 (index 0.75-0.86 indicates moderate depression and a score of 70 or above the depression is considered to be severe [14].

To assess QoL, the 36-Item Short Form Survey (SF-36) consisting of items evaluating 8 subscales which are: physical functioning (PF), physical role (PF), bodily pain (BP), general health (GH), vitality (V), social functioning (SF), mental health (MH) and emotional role (ER) [15]. The more the score is over 50 the better the QoL.

### Statistical analysis

Descriptive statistics (n=frequency, %=percentage) were used to assess the levels of anxiety and depression of healthcare professionals during the COVID-19 period. Both parametric (t-test and one-way ANOVA) and non-parametric tests (Kruskall-Wallis), depending on the homogeneity of variance test, were used to evaluate the differences in the results of the dependent variable of a) mental health (anxiety/depression) with socio-demographic variables and b) the correlation of summary scales of physical health (physical component summary) with gender, specialty and current exhaustion compared to the pre-COVID-19 era. For the power analysis based on the results of the study (post hoc) the following were applied: Type power analysis (Post hot compute achieved power – given a, sample size, and effect size). Effect size (determine n1=n2), a err prob=0.05 = power (1-b err prob). For the effect sizes to determine at which extent a dependant variable affects the dependant one (2 groups) for the t-test we used Cohen’s d and for the One Way ANOVA $\eta^{2}=\frac{\text{ SSbetween }}{\text{ SStotal }}$, and $\eta^{2}=\frac{H-k+1}{N-\kappa }$for Kruskal-Wallis. The Pearson’s correlation coefficient r was used to assess the relationship between anxiety and depression with all other variables, and Tukey’s HSD correlation to test the effect of multiple trials. The scores for the eight dimensions of SF-36 were calculated following the instructions and algorithms of the questionnaire developer. The confidence intervals were set at 95% and the level of significance at p=0.05. For the statistical analysis the statistical package IBM SPSS v.22.0 was used.

## Results

### Reliability of the questionnaires

In the present study, Cronbach’s alpha for SAS was equal to 0.818, for SDS 0.842 and for SF-36 0.709.

### Demographic, professional and COVID-19 related factors and psychological status

**Fig. 1:**
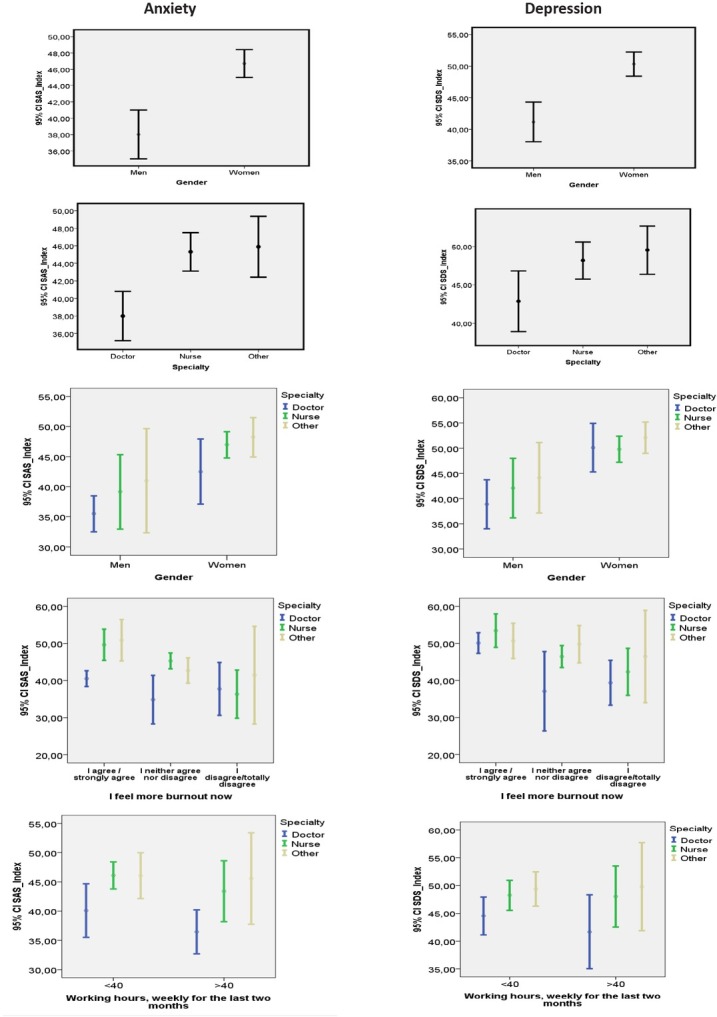
Correlation between SAS and SAS with demographics and other factors & anxiety, depression, physical component summary compared to working hours, weekly for the last two months (average)

As shown in Table 1 and Figure 1 the majority of participants were women with more than 15 years of professional experience. More than half worked between 21-40 hours per week and 42.2% worked two weekends a month. When asked whether they think they had been exposed to COVID-19 half of the participants responded affirmatively. Regarding the respondents’ psychological status, although the majority experienced normal stress levels, a 4.8% experienced moderate levels and a 0.7% severe. As for depression, mild depressive symptoms were reported by 34.7%, moderate by 10.2% and severe by 1.4%.

**Table 1:** Sample characteristics, COVID-19 related factors and psychological status

	N (%)
Gender	
Men	48 (32.7)
Women	99 (77.3)
Specialty	
Medical Doctor	31(21.1)
Medical Nurse	85(57.8)
Other	31(21.1)
Education	
Secondary Higher Education	3(2)
Tertiary Education	87(59.2)
Postgraduate studies	38(25.9)
Doctoral studies	19(12.9)
Marital status	
Unmarried	33(22.4)
Married	100(68.0)
Divorced	14(9.5)
Years of working	
<1	5(3.4)
1-5	14(9.5)
6-10	27(18.4)
11-15	27(18.4)
>15	74(50.3)
Weekly working hours for the last two months	
<10	6(4.1)
11-20	1(7)
21-40	86(58.5)
41-60	48(32.7)
>61	6(4.1)
Word during weekends in the last two months	
Never	38(25.9)
Every two weeks	62(42.2)
Every week one day of the weekend	29(19.7)
Every week on both days	18(12.2)
Department	
Closed section (unit)	78(53.1)
Open ward (Clinic, Health Center)	69(46.9)
Previous medical history	
My health is in good condition	86.4(86.4)
I have a chronic illness	11.6(11.6)
I’m dealing with a psychiatric illness	2.0(2)
Contact with COVID-19	
Agree	74(50.3)
Disagree	14(9.5)
Not sure	59 (40.1)
Feelings of exhaustion compared to pre-COVID-19 era	
Agree/totally agree	54(36.7)
Neither agree nor disagree	63(42.9)
Disagree/totally disagree	30(20.4)
Worries about getting infected	
Agree/totally agree	81(55.1)
Neither agree nor disagree	43(29.3)
Disagree/totally disagree	23(15.6)
Worries that the family will be infected	
Agree/totally agree	104(70.7)
Neither agree nor disagree	20(13.6)
Disagree/totally disagree	22(15)
Worries that the whole situation will last for a long time	
Agree/totally agree	94(63.9)
Neither agree nor disagree	35(23.8)
Disagree/totally disagree	18(12.2)
Considerations of resigning due to COVID-19	
Agree	3(2)
Disagree	135(91.8)
Not sure	9(6.1)
Satisfaction with the protection provided	
Agree	54(36.7)
Disagree	33(22.4)
Not sure	60(40.8)
SAS	
Absence	104 (70.7)
Mild	35 (23.8)
Moderate	7 (4.8)
Severe	1 (0.7)
SDS	
Absence	79 (53.7)
Mild	51 (34.7)
Moderate	15 (10.2)
Severe	2 (1.4)

SAS: Zung Self-Rating Anxiety Classification, SDS: Zung Self-Rating Depression Classification

Correlating SAS and SDS with other variables, as shown in Table 2, a statistical significance was found between men and women healthcare professionals SAS (p=0.001), with large effect sizes (d= 0,85) & large effect power analysis 99%, SDS (p=0.001),with large effect sizes (d= 0,84) & statistical power 99%, with the latter experiencing higher levels of anxiety and depression. Similar statistical significance is observed in the specialty in SAS (p=0.001), with medium to large effect sizes (d= 0,95) & statistical power 95% and in SDS (p=0.001), with large effect sizes (d= 0,095) & statistical power 95%. As far as the specialty is concerned, doctors experienced lower levels of both stress and depression (p=0.001 & 0.050 respectively) compared to nurses (p=0.004) and other professionals (p=0.040). Anxiety and depression were associated with exhaustion. The more exhausted the healthcare professionals felt, SAS (p=0.001), with large effect sizes (d= 0,1) & statistical power 96% και στη SDS (p=0.001), with large effect sizes (d= 0,13) & statistical power 99%, the higher their levels of anxiety and depression were (p=0.023 & p=0.003 respectively) compared to those with a neutral attitude (p=0.023 & 0.003 respectively) and those who felt no exhaustion (p=0.001 & 0.001 respectively). The effect sizes (gender, specialty, burnout) is large to medium with the result of a large power analysis of the results showing that the conclusion can be generalized. Based on the odds ratio (OR 5.357, 95% CI, 1.95-14.72: p=0.001) women were 5.4 times at more risk of anxiety than men and 3.4 times at more risk for depression (OR 3.365, 95% CI, 1.59-7.12: p=0.002).

**Table 2:** Correlation between SAS and SDS with demographics and other factors & anxiety, depression, physical component summary compared to working hours, weekly for the last two months (average)

			Mean	SD	p-value	Statistical method used Effect size Statistical power
Gender	Anxiety	Men	38.02	10.26	0.001*	T-Test
						Effect size
		Women	46.73	8.52		d= 0.85^a^
						Power(1-β err prob)= 0.99
	Depression	Men	41.17	10.83	0.001*	T-Test
						Effect size
		Women	50.32	9.56		d= 0.84^a^
						Power(1-β err prob)= 0.99

Specialty	Anxiety	Doctor	45.79**		0.001*	Kruskal-Wallis Test
		Nurse	80.44**			Due to Test of Homogeneity of Variances=,029
		Other	84.56**			Effect size **η**^2^=0.095^c^
						Power(1-β err prob)= 0.95
	Depression	Doctor	42.86	10.74	0.001*	ANOVA
						Effect size **η**^2^=0.095^b^
		Nurse	48.16	11.27		Power(1-β err prob)= 0.95
		Other	49.52	8.60		

I feel more exhausted	Anxiety	Agree/ strongly agree	88.06**		0.001*	Kruskal-Wallis Test Due to Test of Homogeneity of Vari
now compared		neither agree nor disagree	74.01**			ances=0.002 Effect size
to before Covid-19		Disagree/strong- ly disagree	48.67**			**η**^2^=0.1^a^ Power(1-β err prob)= 0.96
	Depression	Agree/ strongly agree	52.03	9.75	0.001*	ANOVA Effect size
		neither agree nor disagree	45.75	10.53		**η**^2^=0.13^a^ Power(1-β err prob)= 0.99
		Disagree/strongly disagree	42.17	10.36		

Anxiety	Working hours, weekly for the	<40	45,26	8,82	0,028*	T-Test Effect size
	last two months (average)	>40	41,53	11,39		d= 0.33^d^ Power(1-β err prob)= 0.48
Depression	Working hours, weekly for the	<40	47,96	9,23	0,404	ANOVA Effect size
	last two months (average)	>40	46,25	13,20		d= 0.13(small) Power(1-β err prob)= 0.12
Physical Component Summary	Working hours, weekly for the last two months	<40	67,43	12,44	0,008*	ANOVA Effect size d= 0.44^c^
	(average)	>40	73,97	15,01		Power(1-β err prob)= 0.73

(*Statistically significant. **Mean Rank, Effect size (^a^large,^b^medium^, c^medium to large, ^d^small to medium, ^e^small), SAS: Zung Self-Rating Anxiety Classification, SDS: Zung Self-Rating Depression Classification, SD: Standard Deviation)

Anxiety and depression were studied in relation to the levels of burnout which healthcare workers experienced before COVID-19 and during COVID-19 phase. It was found that during COVID-19 stress (p=0.025) and depression (p=0.003) were at higher levels than the pre-COVID-19 period.

Those working under 40 hours experience more stress and have less quality of life in the concise scale of physical health with a statistical significance at p<0.5. This can be attributed to the fact that those working in the HC reported less working hours and absence of contact with COVID-19 patients. In the anxiety scale the effect sizes are small to medium with power analysis at 48% and thus cannot be generalized. In the concise scale of physical health the effect sizes are medium to large with strong power analysis at 73%.

### Quality of Life

Comparing the scores of the sub-scales of SF-36 of the present study to the findings of a first survey of Tountas et al in 2003 [16], it can be observed that health-care workers’ QoL during COVID-19 has declined, as shown in Table 3.

**Table 3:** Rating of Qol parameters

	1^st^ Survey (16)	Current survey
Physical (PF) Functioning	84.2	82.07
Physical Role (PR)	75.7	62.09
Bodily Pain (BP)	74.4	67.78
General Health (GH)	69	67.38
Vitality (VT)	63.5	61.63
Social Functioning (SF)	69.5	61.20
Mental Health (MH)	74.1	65.30
Emotional Role (ER)	66.6	66.09

QoL: Quality of Life

**Fig. 2:**
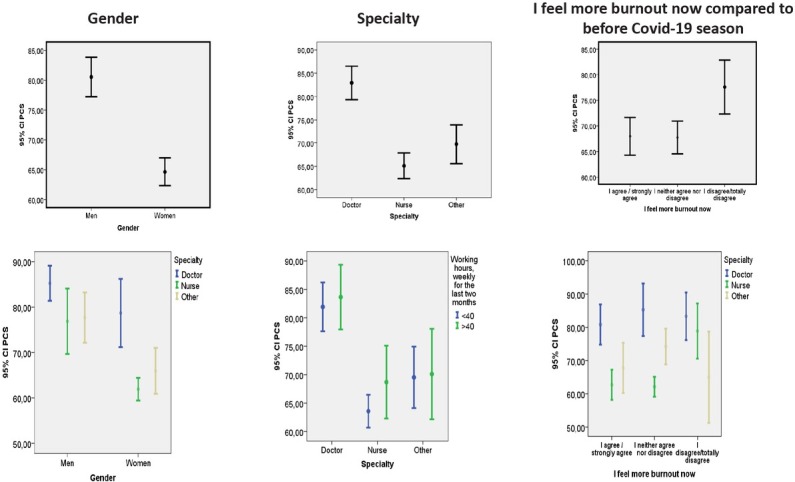
Association of summary scales of Physical Component Summary with gender, specialty and burnout at present, compared to before COVID-19 era

Table 4 and Figure 2 show the correlations of the Physical Component Summary (PCS) with the gender, specialty and burnout. It was found that men have better physical health than women (p=0.001), with large effect sizes (d=1.36; power = 99%). Doctors have a better PCS than nurses (p=0.001) and other healthcare professionals (p=0.001), with large effect sizes (d=0.26; power = 99%). The participants who feel more physically burnout during COVID-19 compared to the era prior the pandemic have worse physical health than those who do not experience more exhaustion during the pandemic (p=0.005).

**Table 4:** Association of summary scales of Physical Component Summary with gender, specialty and burnout at present, compared to before COVID-19 era

		Mean	SD	p-value	Statistical methods Effect size Statistical power
Gender	Male	80.53	11.45	0.001*	T-Test
	Female	64.64	11.66		d= 1.36^a^
					Power(1-β err prob)= 0.99

Specialty	Doctor	82.92	9.75	0.001*	ANOVA
	Nurse	65.09	12.74		Effect size
	Else	69.74	11.46		d= 0.26^a^
					Power(1-β err prob)= 0.99

I feel more burnout now compared to before Covid-19 season	I agree / strongly agree I neither agree nor disagree	67.96 67.74	13.49 12.70	0.002*	ANOVA
					Effect size
					d= 0.082^c^
					Power(1-β err prob)= 0.90
	I disagree/totally disagree	77.58	14.08		

* Statistically important, SD: Standard Deviation, Effect size (^a^large,^b^medium^,^ cmedium to large)

As shown in Table 5, anxiety and depression were negatively correlated with 6 sub-scales of SF-36. More specifically, the more the anxiety and depression increase, the more the physical functionality, physical pain, general health, vitality, mental health and emotional role decrease.

**Table 5:** Anxiety/Depression effect on SF-36 parameters

		PF	PR	BP	GH	VT	SF	ER	MH
SAS	Pearson R	-,541^**^	,026	-,563^**^	-,515^**^	-,616^**^	-,069	-,576^**^	-,624^**^
	p-value	,000	,755	,000	,000	,000	,411	,000	,000
SDS	Pearson R	-,450^**^	-,008	-,369^**^	-,440^**^	-,587^**^	-,056	-,482^**^	-,635^**^
	p-value	,000	,922	,000	,000	,000	,506	,000	,000

**. Correlation is significant at the 0.01 level (2-tailed). SF-36: Short Form Survey,PF: Physical Functioning, PR: Physical Role, BP: Bodily Pain, GH: General Health, VT: Vitality, SF: Social Functioning, MH: Mental Health, ER: Emotional Role, SAS: Zung Self-Rating Anxiety Classification, SD: Zung Self-Rating Depression Classification

## Discussion

Assessing the impact of the COVID-19 pandemic on the psychological status and QoL of healthcare professionals in Athens, Greece, it was observed that although the majority of the participants experienced normal stress and depression levels, moderate to severe levels for both disorders were found at a significant percentage. These findings are in agreement with other studies assessing the psychological status of healthcare workers during the COVID-19 pandemic. Temsah et al [17] also found normal anxiety levels (68.2%), followed by mild anxiety (20.8%) and severe for the 2.9%. Results from another study showed that 51.6% of healthcare workers had anxiety symptoms and 64.7% depressive ones [1]. Hu et al [18] reported mild anxiety for 27.15%, moderate for 11.05% and severe for 3.3%.

In the present study, female healthcare workers experienced higher levels of both anxiety and depression. Moreover, women were at greater risk of developing these two mental disorders compared to men. These findings are in agreement with other studies reporting that women tend to experience more anxiety and depression. In particular, Elbay et al [1] found that women were significantly more stressed than men and Ning et al [19] also reported higher anxiety levels in women healthcare workers. Xiao et al [8] reported that working women are 1.6 times more likely to experience anxiety than men in agreement with other studies [20,21], findings which support that the male gender is a protective factor against stress and depression [20].

The participants in this study were found to experience higher anxiety and depression levels during the COVID-19 pandemic compared to the pre-COVID-19 era, especially among women. These findings are consistent with other research reporting significant differences of stress and depression levels before and during the pandemic [22]. The fact that women tend to be more vulnerable to these two disorders during the current pandemic is due to several factors, such as a higher risk of infection, increased family pressure as well as the effects of female hormones. As for women nurses, their crucial role in the management of COVID-19 can justify the increased levels of anxiety and depression they experience. In addition, due to the nature of their job and their close contact with patients on a day-today basis, nurses are at higher risk of infection [19]. Yin et al [23], assessing the stress symptoms between clinical roles, demonstrated that women were more vulnerable than men in exhibiting post-traumatic stress. These findings are supported by another study which also found increased risk of depression and anxiety among women [24].

Another reason leading to increased anxiety among healthcare workers, who utilize emotion suppression strategies, is the contact with COVID-19 patients. It has been supported that due to that, men have less cognitive re-esteem and emotion suppression than women [6]. Moreover, another pattern observed in women can be linked to a societal issue. Women face more difficult conditions due to their social roles, which lead to differentiating professional and family care, avoiding contact with family members [25].

Comparing anxiety and depression levels between nurses and other specialties in the present study, it was observed that doctors experience less anxiety and depression than nurses and other healthcare workers. Similar results were reported by Shechter et al [26], who demonstrated significant differences in acute stress between nurses and doctors (64% vs. 40%, p=<0.001) and in depression (53% vs. 38%, p=0.004). Similarly, Pandey et al [21], comparing doctors and nurses, found that the latter were twice as likely to experience anxiety. Healthcare professionals with inadequate or no personal protective equipment were nearly three times more likely to be stressed and twice more at risk of depression than those working in high-risk areas. Li et al [27], investigating the levels of anxiety among healthcare workers, found that nurses are 1.41 times more likely to experience anxiety than other specialties. Tan et al [28] investigated the stress of healthcare workers (doctors and nurses) and “non-medical” staff (related health professionals, pharmacists, technicians, administrators, employees and maintenance workers) during the COVIC-19 pandemic. They found that the prevalence of anxiety was higher among non-medical healthcare workers. Another study highlighted the statistically significant difference between clinical roles for post-traumatic stress, reporting significant differences between nurses and, qualified doctors and auxiliary staff (p=0.011) [29]. These findings can be explained by the fact that nurses make up the bulk of healthcare staff during an epidemic and undertake most of the tasks related to infectious diseases [18].

The present study did not demonstrate significant differences in stress and depression levels among healthcare professionals in COVID-19-related departments, as demonstrated by Liang et al [11]. However, it was observed that the healthcare professionals do experience more anxiety and depression during the pandemic compared to the pre-COVID-19 era.

Based on the correlations of the sub-scales of SF-36, this study demonstrated a negative correlation between QoL and both anxiety and depression. More specifically, as anxiety and depression increase, physical functionality, physical pain, general health, vitality, mental health and emotional role tend to decrease. It was also found that men have better physical health than women. Tountas et al [16] have also highlighted gender differences and women in particular were reported to have a lower health status than men in all eight sub-scales of SF-36. Huang et al [30], assessing QoL of healthcare professionals, found that women experienced decreased QoL compared to men regarding emotional and cognitive functioning.

## Conclusions

During this COVID-19 period, increased workload, redistribution of tasks and uncharted guidelines for managing the disease result in increased risk of occupational stress and depression negatively affect healthcare professionals’ QoL. The factors which lead to increased levels of stress and depression among healthcare professionals should be recognized, so as to implement strategies and measures to reduce the psychological burden caused by the pandemic and increase the workers wellbeing and productivity.

The findings of this study should be interpreted within its limitations, the main of which is the limited representation of more specialties mainly due to the measures for the protection of public health.
